# Critical Roles of PIWIL1 in Human Tumors: Expression, Functions, Mechanisms, and Potential Clinical Implications

**DOI:** 10.3389/fcell.2021.656993

**Published:** 2021-02-26

**Authors:** Peixin Dong, Ying Xiong, Yosuke Konno, Kei Ihira, Daozhi Xu, Noriko Kobayashi, Junming Yue, Hidemichi Watari

**Affiliations:** ^1^Department of Obstetrics and Gynecology, Hokkaido University School of Medicine, Hokkaido University, Sapporo, Japan; ^2^State Key Laboratory of Oncology in South China, Department of Gynecology, Sun Yat-sen University Cancer Center, Guangzhou, China; ^3^Department of Pathology and Laboratory Medicine, University of Tennessee Health Science Center, Memphis, TN, United States; ^4^Center for Cancer Research, University of Tennessee Health Science Center, Memphis, TN, United States

**Keywords:** piRNA, PIWIL1, HIWI, tumorigenesis, cancer metastasis, prognostic biomarker, EMT, chemoresistance

## Abstract

P-element-induced wimpy testis (PIWI)-interacting RNAs (piRNAs) are a class of small non-coding RNA molecules that are 24–31 nucleotides in length. PiRNAs are thought to bind to PIWI proteins (PIWL1-4, a subfamily of Argonaute proteins), forming piRNA/PIWI complexes that influence gene expression at the transcriptional or post-transcriptional levels. However, it has been recently reported that the interaction of PIWI proteins with piRNAs does not encompass the entire function of PIWI proteins in human tumor cells. PIWIL1 (also called HIWI) is specifically expressed in the testis but not in other normal tissues. In tumor tissues, PIWIL1 is frequently overexpressed in tumor tissues compared with normal tissues. Its high expression is closely correlated with adverse clinicopathological features and shorter patient survival. Upregulation of PIWIL1 drastically induces tumor cell proliferation, epithelial-mesenchymal transition (EMT), invasion, cancer stem-like properties, tumorigenesis, metastasis and chemoresistance, probably via piRNA-independent mechanisms. In this article, we summarize the current existing literature on PIWIL1 in human tumors, including its expression, biological functions and regulatory mechanisms, providing new insights into how the dysregulation of PIWIL1 contributes to tumor initiation, development and chemoresistance through diverse signaling pathways. We also discuss the most recent findings on the potential clinical applications of PIWIL1 in cancer diagnosis and treatment.

## Introduction

P-element-induced wimpy testis (PIWI)-interacting RNAs (piRNAs) are small (24–31 nucleotides), single-stranded non-coding RNAs with 2′-O-methylated at their 3′ ends (Ng et al., 2016; Wang et al., 2019; Yu et al., 2019). According to their origins, piRNAs can be divided into three classes: transposon-derived piRNAs, mRNA-derived piRNAs, and lncRNA-derived piRNAs (Weick and Miska, 2014; Ozata et al., 2019; Guo et al., 2020). Transposon-derived piRNAs are typically transcribed from both genomic strands and silence transposons (Weick and Miska, 2014; Ozata et al., 2019; Guo et al., 2020). MRNA-derived piRNAs originate from the 3′-untranslated regions (3′-UTRs) of mRNAs (Weick and Miska, 2014; Ozata et al., 2019; Guo et al., 2020). Long non-coding RNA (lncRNA)-derived piRNAs often come from intergenic lncRNAs (Weick and Miska, 2014; Ozata et al., 2019; Guo et al., 2020). The biogenesis of piRNAs is involved in two major mechanisms: the primary synthesis mechanism and a secondary amplification pathway (also referred to as the “ping-pong” amplification cycle) (Weick and Miska, 2014; Ozata et al., 2019; Guo et al., 2020). The primary piRNAs are produced through the primary processing pathway, and the abundance of pre-existing piRNAs can be amplified through the “ping-pong” amplification cycle (Weick and Miska, 2014; Ozata et al., 2019; Guo et al., 2020).

PiRNAs are initially discovered in germline (Yu et al., 2019). Studies of animals suggested that silencing transposons in germline tissues is the ancestral function of piRNAs (Weick and Miska, 2014; Ozata et al., 2019; Guo et al., 2020). It was shown that piRNAs silence transposons transcriptionally by silencing transposon loci, as well as post-transcriptionally by triggering degradation of their transcripts (Weick and Miska, 2014; Ozata et al., 2019; Guo et al., 2020). However, many piRNAs expressed in the mammalian testis map to genome-unique sequences, which are not related to transposable elements (Weick and Miska, 2014; Ozata et al., 2019; Guo et al., 2020), indicating that the biological functions of piRNAs may extend beyond transposon control. In humans, more than 35,356 piRNAs have been found (Wang et al., 2019; Yu et al., 2019), and they are expressed in human somatic cells in a tissue-specific manner (Wang et al., 2019; Yu et al., 2019). Recent studies revealed that piRNAs post-transcriptionally regulate gene expression in microRNA (miRNA)-like manner, thereby participating in the pathogenesis of human cancer (Lu et al., 2010; Liu et al., 2019). The aberrant expression of piRNAs has been demonstrated in various cancer types (Cheng et al., 2011; Huang et al., 2013; Chu et al., 2015). Numerous studies have found that dysregulated piRNAs affect cancer hallmarks for tumor initiation and progression (Yu et al., 2019).

The Argonaute protein family members are ∼100 kDa highly basic proteins that are highly conserved between species (Carmell et al., 2002; Wu et al., 2020). Argonaute proteins can be separated according to the sequence into two subclasses: AGO and PIWI (Parker and Barford, 2006). Argonaute proteins regulate gene expression at both transcriptional and posttranscriptional levels by providing anchor sites for small regulatory RNAs (Parker and Barford, 2006; Wu et al., 2020). In contrast to AGO proteins that are ubiquitously expressed and interact with miRNAs and siRNAs, PIWI proteins (PIWIL1, PIWIL2, PIWIL3, and PIWIL4) are mainly expressed in germ cells, but usually absent in somatic tissues (Qiao et al., 2002; O’Donnell and Boeke, 2007). PIWI proteins use piRNAs as sequence-specific guides to form the piRNA-induced silencing complex, resulting in RNA degradation and epigenetic silencing (Tian et al., 2011; Meister, 2013).

The *PIWIL1* (also called HIWI) gene was first discovered in Drosophila (Cox et al., 1998) and fully identified in a human testis cDNA library (Qiao et al., 2002). *PIWIL1* is located on human chromosome 12q24.33 and encodes an 861-amino acid protein. The PIWIL1 protein contains two characteristic protein domains, namely PAZ domain and PIWI domains (Parker and Barford, 2006). In human testis, PIWIL1 was found to be expressed in late-pachytene spermatocytes and round/elongating spermatids (Hempfling et al., 2017), indicating a potential role for PIWIL1 in human spermatogenesis.

Importantly, overexpression of the *PIWIL1* gene is common to many tumor types (Suzuki et al., 2012), and its aberrant overexpression has been associated with tumorigenesis, tumor development and poor prognosis in different tumors (Suzuki et al., 2012; Tan et al., 2015). Growing evidence showed that PIWIL1 tends to exhibit tumor-promoting roles in sustaining tumor cell proliferation and activating invasion and metastasis (Suzuki et al., 2012; Tan et al., 2015). Although the molecular basis underlying the oncogenic functions of PIWIL1 remains largely unknown, PIWIL1 has been recently found to regulate the occurrence and progression of human cancers possibly through piRNA-independent mechanisms (Genzor et al., 2019; Li et al., 2020; Shi et al., 2020). Given that the expression of PIWIL1 is mostly restricted to the testis (Qiao et al., 2002) and broadly elevated in various tumors, PIWIL1 has the potential to be ideal targets for cancer diagnosis and therapy. Here, we review the most recent studies on PIWIL1, including its abnormal expression, cellular functions, mechanisms, along with its potentials as a biomarker for cancer diagnosis, prognosis evaluation, and a molecular target that enables the design of novel therapeutic strategies.

## Dysregulation of PIWIL1 in Tumor

Northern blot analysis of *PIWIL1* mRNA in a series of adult human normal tissues confirmed that *PIWIL1* is expressed abundantly in the testis, but undetectable in the spleen, thymus, prostate, ovary, small intestine, colon tissue and peripheral blood leukocytes (Qiao et al., 2002). Consistent with the proposed tumor-promoting role of PIWL1 during tumorigenesis and tumor progression, PIWIL1 can be overexpressed in many different types of tumor (Table 1), including gastric cancer (Liu et al., 2006; Wang et al., 2012; Gao et al., 2018), soft-tissue sarcoma (Taubert et al., 2007), esophageal squamous cell carcinoma (He et al., 2009), endometrial cancer (Liu et al., 2010b), colon cancer (Li L. et al., 2010; Litwin et al., 2015; Wang H.L. et al., 2015; Sun et al., 2017), cervical cancer (Li S. et al., 2010; Liu et al., 2010a), glioma (Sun et al., 2011), hepatocellular carcinoma (Jiang et al., 2011; Zhao et al., 2012), ovarian cancer (Li S. et al., 2010; Chen et al., 2013), breast cancer (Li S. et al., 2010; Wang D.W. et al., 2014; Cao et al., 2016; Litwin et al., 2018), bladder cancer (Eckstein et al., 2018), and renal cell carcinoma (Stöhr et al., 2019). However, real-time PCR analysis of renal cell carcinoma and non-tumor renal parenchyma tissues found a significant downregulation of *PIWIL1* in renal cell carcinoma tissues (Iliev et al., 2016).

**TABLE 1:** The association between *PIWIL1* expression and clinicopathological factors of tumor.

Cancer type	No.	Method	Expression	Clinical factors					References
	
				Size	Stage/grade	Invasion depth	LN meta/recurrence	Survival	
Gastric cancer	50	IHC	Upregulation	–	–	–	–	–	Liu et al., 2006
Gastric cancer	182	Tissue microarray	Upregulation	–	–	–	–	Poor	Wang et al., 2012
Gastric cancer	120	IHC	Upregulation	–	+	+	+	Poor	Gao et al., 2018
Soft-tissue sarcoma	65	qPCR	–	–	–	–	–	Poor	Taubert et al., 2007
Esophageal squamous cell carcinoma	137	IHC	Upregulation	–	+	–	+	Poor	He et al., 2009
Endometrial cancer	64	IHC	Upregulation	–	–	–	–	–	Liu et al., 2010b
Colon cancer	75	IHC	Upregulation	–	–	–	–	–	Li L. et al., 2010
Colon cancer	178	IHC	Upregulation	–	+	–	+	–	Wang H.L. et al., 2015
Colon cancer	110	IHC	Upregulation	–	+	+	+	Poor	Sun et al., 2017
Colon cancer	72	qPCR	Upregulation	–	+	+	–	–	Litwin et al., 2015
Cervical cancer	59	IHC	Upregulation	–	+	–	+	–	Liu et al., 2010a
Cervical, breast, ovarian and endometrial cancer	–	IHC	Upregulation	–	–	–	–	–	Li S. et al., 2010
Glioma	66	IHC	–	–	+	–	–	Poor	Sun et al., 2011
Hepatocellular carcinoma	92	IHC	Upregulation	–	–	–	+	Poor	Jiang et al., 2011
Hepatocellular carcinoma	336	IHC	Upregulation	+	–	–	+	Poor	Zhao et al., 2012
Ovarian cancer	20	IHC	Upregulation	–	–	–	–	–	Chen et al., 2013
Breast cancer	240	Western blot	Upregulation	+	+	–	+	–	Wang D.W. et al., 2014
Breast cancer	27	qPCR/western blot	Upregulation	–	–	–	–	Poor	Cao et al., 2016
Breast cancer	101	IHC	Upregulation	–	–	–	–	–	Litwin et al., 2018
Bladder cancer	95	IHC	–	–	–	–	+	Poor	Eckstein et al., 2018
Renal cell carcinoma	610	Tissue microarray/IHC	–	–	+	+	+	Poor	Stöhr et al., 2019
Renal cell carcinoma	57	qPCR	Downregulation	–	–	–	–	Better	Iliev et al., 2016

**LN meta, lymph node metastasis.**

**FIGURE 1:**
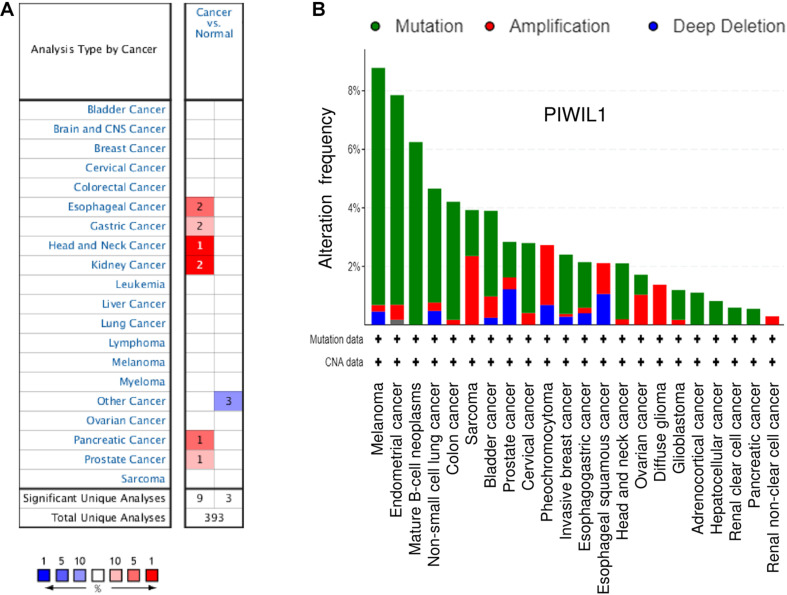
The expression of *PIWIL1* in different types of tumors. **(A)** Comparison of *PIWIL1* mRNA levels in different tumors and in normal tissues according to the Oncomine database (https://www.oncomine.org). Red: upregulation; blue: downregulation. **(B)** Analysis of genetic alterations in *PIWIL1* in human tumor tissues using the Cancer Genome Atlas (TCGA) data retrieved from the cBioPortal database (https://www.cbioportal.org). Data are represented as a stacked histogram plot. Colors represent different types of alterations as indicated in the legend. Shown is the “Cancer Types Summary” where green indicates mutation, red indicates amplification, and blue indicates deletion.

The mRNA expression of *PIWIL1* in different types of tumors was explored using the Oncomine database^1^. Twelve studies showed significant differences in *PIWIL1* mRNA expression between tumor and normal tissues (Figure 1A). The expression levels of *PIWIL1* were significantly increased in esophageal cancer, gastric cancer, head and neck cancer, kidney cancer, pancreatic cancer, and prostate cancer tissues compared with respective normal tissues (Figure 1A).

When looking at all cancer types in the TCGA data sets from the cBioPortal database^2^, the *PIWIL1* gene is amplified in many tumors, in line with a tumor-promoting role (Figure 1B). Amplifications are more frequent in diffuse glioma, sarcoma, pheochromocytoma, ovarian cancer, bladder cancer, cervical cancer, esophageal squamous cell carcinoma, and renal non-clear cell carcinoma (Figure 1B). These results suggest that *PIWIL1* dysregulation is frequently occurring in human tumor tissues and *PIWIL1* might serve as a novel biomarker in several malignancies.

In melanoma, endometrial cancer, mature B-cell neoplasms, non-small cell lung cancer, colon cancer, bladder cancer, invasive breast cancer, esophagogastric adenocarcinoma and head and neck cancer, *PIWIL1* is often mutated (Figure 1B). Consistently, data from the IntOGen database^3^ revealed that 362 *PIWIL1* mutations were found in 28,076 samples of various cancers, including a range of mutation types (such as missense and truncating mutations).

## Implications of PIWIL1 Expression in Cancer Diagnosis and Prognostic Evaluation

Dysregulation of PIWIL1 occurs in a broad range of human cancers and is often associated with adverse clinicopathological features and shorter survival of cancer patients. For example, PIWIL1 expression is progressively increased in normal gastric tissues, atrophic gastritis, intestinal metaplasia and gastric cancer tissues (Liu et al., 2006), holding diagnostic potentials for improving early gastric cancer detection. Similarly, positive PIWIL1 expression in normal tissues, colonic adenoma and colon cancer was 11.1% (5/45), 53.7% (22/41), and 80.4% (74/92), respectively (Wang H.L. et al., 2015). Also, a study of normal cervical tissues, high-grade squamous intraepithelial lesions (HSILs) and cervical cancer samples showed a significantly higher frequency of PIWIL1 protein expression in HSILs and cervical cancer tissues when compared with that in the normal cervical epithelium (Liu et al., 2014). Another study observed that the protein levels of PIWIL1 increase in a stepwise manner in normal endometrium, endometrial atypical hyperplasia and endometrial cancer tissues (Chen et al., 2015a). Overexpression of PIWIL1 is an early event in colon carcinogenesis, since it is significantly upregulated from the earliest stages (I and II) of colon cancer progression compared to normal colon tissues (Sellitto et al., 2019). These studies indicate that PIWIL1 levels could be correlated with tumor progression and may be used to facilitate early cancer diagnosis.

High PIWIL1 expression was associated with higher histological grade and advanced tumor stage in different types of tumors, such as esophageal cancer (He et al., 2009), glioma (Sun et al., 2011), breast cancer (Wang D.W. et al., 2014), and renal cell carcinoma (Stöhr et al., 2019), linking its expression to de-differentiation and the progression of the tumor phenotypes. For several cancers, the expression of PIWIL1 expression at different stages of cancer has been examined (He et al., 2009; Liu et al., 2010a, 2014; Chen et al., 2015a; Wang H.L. et al., 2015; Sun et al., 2017; Stöhr et al., 2019). For instance, in esophageal cancer (He et al., 2009), colon cancer (Wang H.L. et al., 2015; Sun et al., 2017), cervical cancer (Liu et al., 2010a, 2014), renal cell carcinoma (Stöhr et al., 2019), and endometrial cancer (Chen et al., 2015a), PIWIL1 levels were significantly increased in late-stage tumors than in early stage tumors. Moreover, an induced expression of PIWIL1 has been linked to lymph node metastasis in patients with gastric cancer (Wang et al., 2012; Gao et al., 2018), colon cancer (Sun et al., 2017), hepatocellular carcinoma (Jiang et al., 2011; Zhao et al., 2012), breast cancer (Wang D.W. et al., 2014), bladder cancer (Eckstein et al., 2018), renal cell carcinoma (Stöhr et al., 2019), and endometrial cancer (Chen et al., 2015a). These findings demonstrate that high PIWIL1 expression might be considered as a useful marker for an aggressive phenotype of several malignancies.

Furthermore, PIWIL1 was found to be a poor prognostic factor in several tumors, including gastric cancer (Wang et al., 2012; Gao et al., 2018), soft-tissue sarcoma (Taubert et al., 2007), esophageal squamous cell carcinoma (He et al., 2009), colon cancer (Sun et al., 2017), glioma (Sun et al., 2011), hepatocellular carcinoma (Jiang et al., 2011; Zhao et al., 2012), breast cancer (Cao et al., 2016), bladder cancer (Eckstein et al., 2018), and renal cell carcinoma (Stöhr et al., 2019). However, a study in patients with renal cell carcinoma showed that high PIWIL1 expression is correlated with better prognosis (Iliev et al., 2016).

**FIGURE 2:**
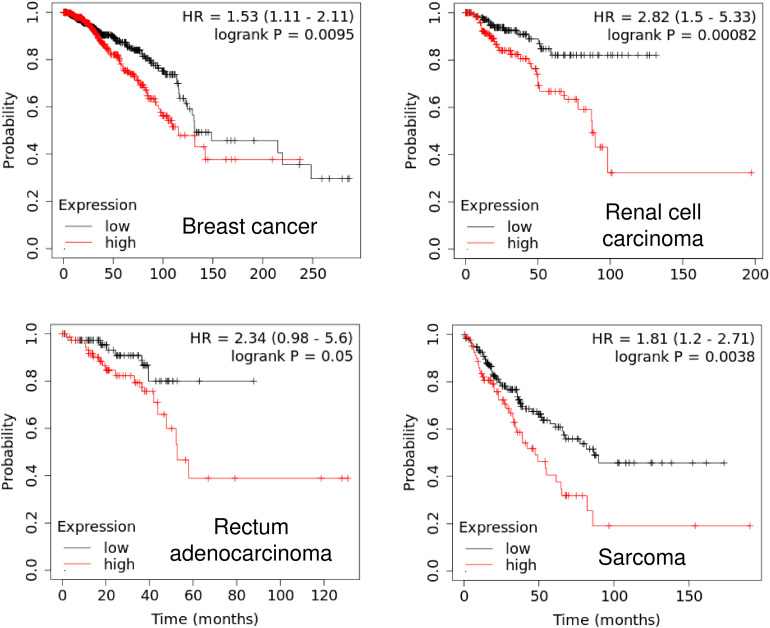
High PIWIL1 expression predicts poor prognosis in patients with tumors. The probability of overall survival in patients with high or low *PIWIL1* expression in different tumors was assessed using the KM plotter database (http://kmplot.com).

Our Kaplan-Meier analysis using the KM plotter database^4^ established a close association of *PIWIL1* expression with unfavorable patient survival. Higher *PIWIL1* mRNA expression is significantly correlated to worsen overall survival for patients with breast cancer, renal cell carcinoma, rectum adenocarcinoma and sarcoma (Figure 2). Thus, the identification of aberrant PIWIL1 expression in tumor tissues might be useful in cancer diagnosis as well as in prognostic evaluation.

## Mechanisms of *PIWIL1* Dysregulation in Tumor

**FIGURE 3:**
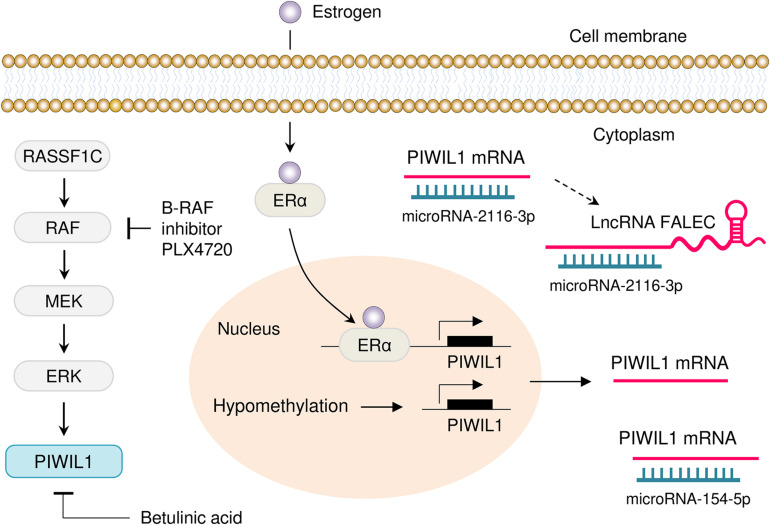
Mechanisms of PIWIL1 dysregulation in tumor. Several mechanisms that drive overexpression of PIWIL1 have been discovered, including activation of the RASSF1C/MEK/ERK pathway, hypomethylation of the *PIWIL1* promoter and enhanced binding of ERα to the *PIWIL1* promoter. Also, PIWIL1 is targeted by miR-154-5p and miR-2116-3p in tumor cells. Moreover, lncRNA FALEC indirectly induces PIWIL1 expression by sponging miR-2116-3p. Inhibition of B-RAF with PLX4720 (a selective B-RAF inhibitor) and treatment with Betulinic acid (a plant secondary metabolite isolated from birch trees), causes downregulation of PIWIL1 in cancer cells.

Multiple transcriptional and post-transcriptional mechanisms by which PIWIL1 is inappropriately overexpressed in tumors have been summarized (Figure 3). Activation of the RASSF1C/MEK/ERK pathway has been shown to induce PIWIL1 expression in non-small cell lung cancer cells (Reeves et al., 2012). In addition, aberrant promoter DNA hypomethylation is one of the major mechanisms for PIWIL1 overexpression in lung cancer (Xie et al., 2018) and endometrial cancer (Chen et al., 2020). In endometrial cancer cells, estrogen was shown to enhance the transcription of *PIWIL1* by facilitating the binding of the ERα to the *PIWIL1* promoter (Chen et al., 2020). A positive correlation between HPV16 E7 and PIWIL1 was detected in cervical cancer tissues, although the related mechanism has not yet been described (Liu et al., 2010a).

Genome projects have shown that functional products encoded by the genome are not limited to proteins, but include a large number of biologically meaningful non-coding RNAs, such as miRNAs, circular RNAs (circRNAs) and lncRNAs (Anastasiadou et al., 2018; Yamamura et al., 2018). MiRNAs are known to target 3′-UTRs in mRNAs, thereby silencing gene expression at the post-transcriptional level (Anastasiadou et al., 2018; Yamamura et al., 2018; Xu et al., 2020). MiRNAs also interact with circRNAs and lncRNAs to regulate their stability (Anastasiadou et al., 2018; Yamamura et al., 2018). Owing to their functions in the regulation of gene expression, non-coding RNAs regulate multiple biological processes, such as cancer (Anastasiadou et al., 2018; Yamamura et al., 2018; Xu et al., 2020). The expression of PIWIL1 could be regulated by different miRNAs at the post-transcriptional level. MiR-154-5p directly targets PIWIL1 and decreases its expression in glioblastoma (Wang et al., 2017) and glioma (Zhou et al., 2020). In addition to miRNAs, lncRNA FALEC has been implicated in the regulation of PIWIL1 expression in colon cancer cells (Jiang et al., 2020). This study demonstrated that depletion of FALEC by shRNA could significantly decrease the proliferation, migration, invasion, angiogenesis and tumorigenesis of colon cancer cells, whereas these inhibitory effects were largely counteracted by ectopic PIWIL1 overexpression (Jiang et al., 2020). Furthermore, lncRNA FALEC induces PIWIL1 expression by serving as a molecular sponge for miR-2116-3p, which directly binds to the 3′-UTR of *PIWIL1* mRNA (Jiang et al., 2020).

## Role of PIWIL1 in Tumorigenesis and Tumor Progression and Possible Mechanisms

**FIGURE 4:**
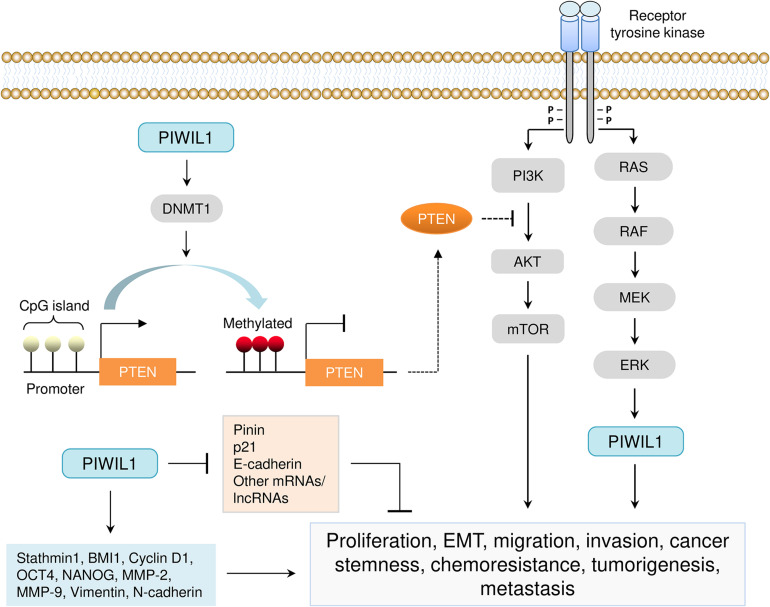
Aberrant expression of *PIWIL1* mediates tumorigenesis and progression. PIWIL1 controls the expression of numerous downstream targets (such as PTEN, DNMT1, Pinin, p21, E-cadherin, BMI1, Stathmin1, Cyclin D1, OCT4, NANOG, MMP-2, MMP-9, Vimentin, and N-cadherin) involved in biological processes that are crucial for PIWIL1-dependent tumor promotion (including cell proliferation, EMT, migration, invasion, cancer stem-like properties, chemoresistance, tumorigenesis and metastasis). PIWIL1 epigenetically silences the expression of PTEN (a novel inhibitor of the PI3K/AKT pathway) by promoting DNA hypermethylation of the promoter of *PTEN*.

Extensive studies have uncovered an important oncogenic role for PIWIL1 in cancer tumor initiation, progression and metastasis (Liu et al., 2006, 2014; Siddiqi et al., 2012; Wang et al., 2012, 2017; Zhao et al., 2012; Liang et al., 2013; Wang D.W. et al., 2014; Wang X. et al., 2014; Chen et al., 2015a; Li et al., 2015, 2020; Xie et al., 2015, 2018; Yang et al., 2015; Cao et al., 2016; Araújo et al., 2018; Gao et al., 2018; Jiang et al., 2020; Shi et al., 2020; Zhou et al., 2020; Table 2 and Figure 4). Many studies have demonstrated that PIWIL1 drives tumorigenesis, malignant progression and metastasis by promoting cell migration, invasion, epithelial-mesenchymal transition (EMT), stem-like properties, tumorigenesis and metastasis, while inhibiting apoptosis.

**TABLE 2:** Roles, cellular functions and underlying mechanisms of PIWIL1 in tumor cells.

Tumor type	Role	Function	Mechanism	References
Pancreatic cancer	Oncogene	Proliferation, migration, invasion, tumorigenesis, metastasis	Acting as a co-activator of APC/C to degrade the cell-adhesion protein Pinin	Li et al., 2020
Gastric cancer	Oncogene	Proliferation, migration, tumorigenesis, metastasis	Forming a complex with UPF1, UPF2 and SMG1 to degrade its target mRNAs and lncRNAs	Shi et al., 2020
Gastric cancer	Oncogene	Proliferation	Cell cycle regulation	Liu et al., 2006
Gastric cancer	Oncogene	Proliferation, migration, invasion	–	Gao et al., 2018
Hepatocellular carcinoma	Oncogene	Proliferation, invasion	–	Zhao et al., 2012
Breast cancer	Oncogene	Proliferation	–	Wang D.W. et al., 2014
Breast cancer	Oncogene	Apoptosis, cell cycle arrest	Possibly regulating transforming growth factor-β receptors and cyclin-dependent kinases	Cao et al., 2016
Cervical cancer	Oncogene	Sphere formation, tumorigenesis, resistance to cisplatin	Possibly increasing OCT4, NANOG and BMI1 expression	Liu et al., 2014
Endometrial cancer	Oncogene	EMT, stem-like properties	Decreasing E-cadherin expression, while the increasing Vimentin, N-cadherin, CD44 and ALDH1 expression	Chen et al., 2015a
Lung adenocarcinoma	Oncogene	Proliferation, migration, invasion	–	Xie et al., 2018
Glioblastoma	Oncogene	Proliferation, apoptosis, invasion	–	Wang et al., 2017
Glioma	Oncogene	Proliferation, invasion	–	Zhou et al., 2020
Colon cancer	Oncogene	Proliferation, migration, invasion, angiogenesis, tumorigenesis	–	Jiang et al., 2020
Lung cancer stem cells	Oncogene	Tumorigenesis	–	Liang et al., 2013
Gastric cancer	Oncogene	Migration, invasion	Possibly regulating several genes involved in migration and invasion processes	Araújo et al., 2018
Sarcoma	Oncogene	Proliferation, tumorigenesis	Promoting global DNA methylation	Siddiqi et al., 2012
Colon cancer	Oncogene	Proliferation	Promoting global DNA methylation	Yang et al., 2015
Glioma	Oncogene	Proliferation, migration, invasion, tumorigenesis	Possibly increasing Cyclin D1, MMP-2 and MMP-9 expression, whereas decreasing p21 expression	Wang X. et al., 2014
Hepatocellular carcinoma	Oncogene	Proliferation, migration	–	Xie et al., 2015
Hepatocellular carcinoma, cervical cancer	Oncogene	Proliferation, migration, invasion	Inducing STMN1 expression	Li et al., 2015
Chronic myeloid leukemia	Tumor suppressor	Proliferation, migration, tumorigenesis	Decreasing MMP-2 and MMP-9 expression	Wang Y. et al., 2015
Acute myeloid leukemia	Tumor suppressor	Proliferation, apoptosis	–	Sharma et al., 2001
Glioblastoma	Oncogene	Apoptosis, senescence, stem-like properties	Decreasing BTG2 and FBXW7 expression, whereas increasing c-MYC, Olig2 and Nestin expression	Huang et al., 2021

Of note, controversies exist regarding the underlying mechanisms of PIWIL1 in tumors. In colon cancer cells, several piRNAs seem to be loaded into a complex consisting of PIWIL1 and specific mRNAs (Sellitto et al., 2019), indicating that the formation of PIWIL1/piRNA complex might exert biological roles in colon cancer. It will be of great interest to understand whether PIWI proteins could utilize piRNAs as targeting guides to influence the stability of specific mRNA targets in tumor cells (Mai et al., 2018). However, recent observations that PIWIL1 does not associate with piRNAs in pancreatic (Li et al., 2020) and gastric cancer cells (Shi et al., 2020) supported the hypothesis that upregulated PIWIL1 protein probably functions in a piRNA-independent manner in cancer cells.

In contrast to these oncogenic activities, previous studies suggested that PIWIL1 may have a tumor suppressor function in some cancer types, including chronic myeloid leukemia and acute myeloid leukemia (Sharma et al., 2001; Wang Y. et al., 2015). This tumor suppressor activity is thought to be controlled by some signaling pathways that decrease the expression of MMP-2/MMP-9 and increase cell apoptosis.

## Regulation of Cell Proliferation, Invasiveness, Tumorigenesis and Metastasis

A previous study using co-immunoprecipitation and next-generation sequencing analysis demonstrated that RNA fragments interacting with PIWIL1 were indistinguishable from background pull-down (Genzor et al., 2019). This provided the initial evidence for eliminating the formation of functional piRNA/PIWIL1 complexes in a colon cancer cell line COLO205 (Genzor et al., 2019). Consistently, a recent study revealed that piRNA expression was not detectable in several PIWIL1-expressing cancer cell lines, and co-immunoprecipitation assays failed to detect the association of PIWIL1 with small RNAs in pancreatic, breast, colon and gastric cancer cells that express PIWIL1 (Li et al., 2020). Comprehensive functional and mechanistic studies showed that, even in the absence of piRNA loading, PIWIL1 could still promote pancreatic cancer metastasis by acting as a co-activator of the anaphase-promoting complex/cyclosome to degrade a critical cell adhesion-related protein, Pinin (Li et al., 2020). Furthermore, a piRNA-independent mechanism has been proposed to account for the oncogenic functions of PIWIL1 in gastric cancer cells (Shi et al., 2020). Their results suggested that *PIWIL1* can significantly boost cell proliferation, migration, tumorigenesis and metastasis by forming a complex with UPF1, UPF2, SMG1 and other components to degrade mRNAs and lncRNAs with tumor suppressor potential (Shi et al., 2020).

Another major mechanism by which PIWIL1 promotes endometrial cancer progression might be through the induction of DNA methylation at *PTEN* CpG islands (Chen et al., 2015b). PIWIL1 causes epigenetic silencing of *PTEN* gene via the upregulation of DNA methyltransferase DNMT1 in endometrial cancer cells (Chen et al., 2015b). Using a mouse model, it was demonstrated that overexpression of PIWIL1 in sarcoma cells was sufficient to promote tumorigenesis, possibly through inducing global DNA methylation (Siddiqi et al., 2012). In addition, PIWIL1 overexpression with an adenovirus vector significantly increases the proliferation of colon cancer cells by increasing global DNA methylation levels (Yang et al., 2015).

## Regulation of EMT, Cancer Stem Cell-Like Properties and Drug Resistance

In cervical cancer, PIWIL1 has been associated with enhanced sphere formation and tumorigenesis, increased resistance to cisplatin, and elevated expression of several stem cell self-renewal-genes (*OCT4*, *NANOG* and *BMI1*) (Liu et al., 2014). PIWIL1 can drive EMT in endometrial cancer cells by upregulating the expression of Vimentin and N-cadherin and by decreasing E-cadherin expression (Chen et al., 2015a). This study also suggested that the pro-cancer stem cell activities of PIWIL1 might be mediated by the induction of two stem cell-related genes (*CD44* and *ALDH1*) (Chen et al., 2015a). Recently, it was shown that PIWIL1 is enriched in glioma stem-like cells (GSCs) and silencing PIWIL1 in GSCs impaired their self-renewal and triggered senescence or apoptosis (Huang et al., 2021). PIWIL1 knockdown strongly increased the expression of BTG2 and FBXW7, but reduced the levels of c-MYC, Olig2 and Nestin in GSCs (Huang et al., 2021). These results supported that PIWIL1 is important for multiple aspects of tumor biology, including EMT-driven metastatic growth, the maintenance of cancer stem cell-like phenotypes, and resistance to therapeutic agents.

## Targeting PIWIL1 for Cancer Therapy

The potential use of PIWIL1 as a therapeutic target for human cancers has been studied previously (Li et al., 2020; Shi et al., 2020). Several strategies have been developed to target PIWIL1 in tumor cells either directly or indirectly.

RNA interference (RNAi)-mediated suppression of PIWIL1 expression in tumor cells reduced proliferation, migration, invasion, EMT, sphere formation and angiogenesis (Zhao et al., 2012; Wang D.W. et al., 2014; Wang X. et al., 2014; Li et al., 2020; Shi et al., 2020). Inactivation of PIWIL1 in mouse models of pancreatic cancer leads to significant tumor shrinkage and a dramatic reduction in metastatic growth (Li et al., 2020). Knockout of *PIWIL1* using the CRISPR/Cas9 system markedly attenuates the tumor growth of gastric cancer *in vivo* (Shi et al., 2020). Therefore, RNAi and CRISPR/Cas9 techniques can be explored as a potential therapeutic strategy for tumors overexpressing PIWIL1.

Since small molecules that bind directly to PIWIL1 and alter its function have not yet been achieved, targeting the signaling pathways that contribute to PIWIL1 dysregulation has been exploited as new approaches to treat PIWIL1-expressing cancers (Reeves et al., 2012; Herr et al., 2015). One of these pathways is the RAS/RAF/MEK/ERK pathway, and several MEK inhibitors have been developed. For example, PLX4720 is a selective B-RAF inhibitor, and treatment with this drug strongly downregulates the expression of PIWIL1 in colon cancer cells (Herr et al., 2015). Betulinic acid, a plant secondary metabolite isolated from birch trees, was shown to inhibit cell proliferation and reduce the levels of PIWIL1 in gastric cancer and lung cancer (Yang et al., 2010; Reeves et al., 2014). Other targets that have been explored in PIWIL1-expressing tumors include miR-154-5p (Wang et al., 2017; Zhou et al., 2020) and miR-2116-3p (Jiang et al., 2020). Therefore, suppression of PIWIL1 expression via introducing miR-154-5p/miR-2116-3p mimics or downregulating the levels of lncRNA FALEC might be additional strategies in PIWIL1-positive tumors.

The acquisition of EMT and cancer stem-cell properties is a possible mechanistic basis for anti-cancer drug resistance (Shibue and Weinberg, 2017). After knocking down PIWIL1 in cervical cancer cells by shRNA, increased sensitivity to cisplatin was observed (Liu et al., 2014). In endometrial cancer, PIWIL1 has been associated with EMT and cancer stem cell-like characteristics (Chen et al., 2015a). These results suggest that, in the future, it could be useful to combine inhibitors against PIWIL1 with other cytotoxic drugs.

## Perspectives

PIWIL1 has a critical role in the initiation, growth, progression, local and distant invasion, and treatment resistance. However, evidence also supports a tumor suppressor role for PIWIL1 in some cell types. Whether PIWIL1 has a context-dependent function in different cancers, and whether PIWIL1 expression may serve as a biomarker for cancer subtyping and re-classification needs to be explored. Whether genomic mutations in *PIWIL1* gene are associated with induced PIWIL1 expression remains unclear, and the functional consequences of such mutations in tumor cells are not well understood. In addition, despite the recent advances in our understanding of PIWIL1, upstream regulators of PIWIL1 as well as its downstream signaling pathways in human tumors remain largely elusive. Furthermore, it would be better to use PIWIL1 inhibitors as an adjuvant to chemotherapy or other treatments. A deeper understanding of the crosstalk between PIWIL1 and other signaling pathways would be important to design effective therapeutic strategies that could sensitize PIWIL1-expressing tumor cells to chemotherapeutic agents or targeted therapies.

## Conclusion

In sum, PIWIL1 has proven its tumor-promoting roles in various aspects of cancer biology. The restricted expression of PIWIL1 in normal adult tissues, and its overexpression in a broad spectrum of malignancies, has led to the consideration of PIWIL1 as an ideal target for cancer diagnosis and treatment.

## Author Contributions

PD wrote the manuscript. All authors contributed to the article and approved the submitted version.

## Conflict of Interest

The authors declare that the research was conducted in the absence of any commercial or financial relationships that could be construed as a potential conflict of interest.

## Footnotes

1 https://www.oncomine.org

2 https://www.cbioportal.org

3 https://www.intogen.org/search

4 http://kmplot.com
